# Divergent survival and viral shedding patterns of red sea bream iridovirus in black sea bream and Japanese sea bass

**DOI:** 10.1111/jfb.70456

**Published:** 2026-04-19

**Authors:** Chae‐yeong Ji, Sung‐Bin Moon, Mun‐Gyeong Kwon, Jae‐Ok Kim, Chan‐Il Park, Kyung‐Ho Kim

**Affiliations:** ^1^ Department of Marine Biology and Aquaculture, College of Marine Sciences Gyeongsang National University Tongyeong Republic of Korea; ^2^ Department of Aquatic Life Medicine, College of Marine Sciences Gyeongsang National University Tongyeong Republic of Korea; ^3^ Aquatic Disease Control Division National Fishery Products Quality Management Service Busan Republic of Korea

**Keywords:** cohabitation, red Sea bream iridovirus, transmission, viral shedding

## Abstract

In open marine aquaculture, rearing diverse fish species in adjacent net pens facilitates the waterborne spread of red sea bream iridovirus (RSIV). This study systematically evaluated the horizontal transmission dynamics of RSIV in black sea bream (BSB, *Acanthopagrus schlegelii*) and Japanese sea bass (JS, *Lateolabrax japonicus*) under immersion and cohabitation conditions. After viral exposure, BSB exhibited high survival rates despite significant viral loads and persistent shedding, effectively acting as an asymptomatic reservoir. Conversely, JS functioned as a highly susceptible host, experiencing 0% survival alongside rapid, high‐level viral replication and dissemination prior to mortality. These divergent epidemiological roles highlight the critical need for species‐specific biosecurity and disease management strategies in shared marine aquaculture zones.

In South Korea, net‐pen aquaculture encompasses a range of commercially important marine fish species, such as black sea bream (*Acanthopagrus schlegelii*), Japanese sea bass (JS, *Lateolabrax japonicus*), red sea bream (*Pagrus major*) and rockfish (*Sebastes schlegelii*), all of which are commonly farmed alongside rock bream (*Oplegnathus fasciatus*) (Kim et al., 2026). These species are often co‐cultured in proximity within the same water areas, creating favourable conditions that facilitate the waterborne spread of viral diseases from highly susceptible hosts to other species (Jahangiri et al., 2022; Ogawa & Timi, 2015). This risk is compounded in net‐pen systems, where pathogens released into the surrounding seawater can persist and circulate, regardless of host mortality.

Among consequential pathogens affecting these farming systems, red sea bream iridovirus (RSIV) is a double‐stranded DNA virus classified as *Megalocytivirus pagrus* 1 (International Committee on Taxonomy of Viruses (ICTV), 2024) that causes high pathogenicity and mortality in the genus *Oplegnathus*, including rock bream. However, RSIV infection in other species, including rockfish, tends to follow a significantly different course, characterised by asymptomatic presentation and or pathogenicity (Jeong et al., 2023). For example, in farmed rockfish in South Korea, massive RSIV‐associated mortality in rock bream cultured in the same water area resulted in no corresponding mortality in rockfish reared in adjacent net pens, with virus presence confirmed only through asymptomatic detection (Kwon et al., 2015). However, most research evaluating RSIV pathogenicity across co‐cultured domestic species has mainly focused on rock bream, red sea bream and rockfish (Kim et al., 2023; Kim et al., 2024; Min et al., 2021). Moreover, despite repeated reports of RSIV detection in black sea bream and Japanese sea bass at South Korean aquaculture facilities (National Fishery Products Quality Management Service (NFQS), 2023), systematic pathogenicity assessments, infection dynamics analyses and horizontal transmission experiments conducted under naturalistic conditions remain extremely limited for both species. Consequently, the epidemiological roles of black sea bream and Japanese sea bass as RSIV hosts remain less understood than those of comparative well‐characterised species, such as rockfish or rock bream, highlighting a knowledge gap. Therefore, in this study, we aimed to systematically evaluate the potential for interspecies horizontal transmission of RSIV under cohabitation conditions by investigating RSIV infection establishment, viral shedding ratio, viral dynamics and mortality patterns in black sea bream and Japanese sea bass.

RSIV genotype II was propagated using spleen homogenates of infected rock bream and inoculated onto monolayers of *P. major* fin cells, as previously described (Kim et al., 2022; Kwon et al., 2020). Culture supernatants exhibiting cytopathic effects at 7 days post‐inoculation were collected and filtered before use. Three fish species were obtained from hatcheries in Gyeongsangnam‐do: rock bream (*n* = 120, 7.8 ± 0.5 cm, 8.0 ± 0.7 g), Japanese sea bass (*n* = 300, 8.8 ± 1.2 cm, 7.5 ± 0.8 g) and black sea bream (*n* = 300, 5.8 ± 0.7 cm, 4.5 ± 1.6 g). All fish were confirmed to be free of bacterial and parasitic infections. For each species, fish were assigned to two experimental groups and immersed in virus suspensions at final concentrations of 10^3^ and 10^5^ copies/mL, respectively, for 4 h at 25°C. After exposure, fish from each treatment group were randomly allocated to two duplicate tanks (30 fish per tank). One tank was used to determine tissue viral loads [*n* = 3, randomly sampled at 1, 4, 7, 14, 21 and 28 days post‐infection (dpi)], whereas the other was used to monitor cumulative mortality and viral shedding kinetics over 30 days. Throughout the experiment, 50% of the rearing water in each tank was replaced daily.

To investigate the horizontal transmission dynamics of RSIV among host species, cohabitation challenge trials were conducted at 25°C. Six donor–recipient pairings were evaluated: JS → BSB, JS → RB, JS → JS, BSB → BSB, BSB → RB, BSB → JS, with JS indicating Japanese sea bass, BSB indicating black sea bream and RB indicating rock bream. Donor fish were intraperitoneally injected with 10^6^ copies per fish and subsequently transferred to new experimental tanks equipped with mesh partitions separating donors from recipients. Thirty healthy (naive) fish were then introduced into the adjacent compartment of each tank. Viral transmission kinetics were evaluated by quantifying tissue viral loads (*n* = 3, sampled at 1, 4, 7, 14, 21, 30 and 40 dpi) and viral shedding into seawater at the corresponding time points.

For tissue analysis, spleens were aseptically excised from fish killed using benzocaine. For seawater analysis, viral concentration was performed using a modified iron flocculation method (Kawato et al., 2016; Kim et al., 2024). Briefly, 500 mL of pre‐filtered seawater was treated with iron chloride and stirred for 1 h at room temperature (20–25°C) to facilitate floc formation. The resulting Fe–RSIV complexes were collected via vacuum filtration. Genomic DNA from spleen tissues and filters was extracted using an AccuPrep Genomic DNA Extraction Kit (Bioneer, South Korea). RSIV genomic copy numbers were quantified using TaqMan‐based quantitative polymerase chain reaction, as previously described (Kim et al., 2022; Table 1).

**TABLE 1:** List of primers and probes used in this study.

Target	Primer/probe	Sequence (5′–3′)	Condition	Expected size	References
Major capsid protein	Meg 1041F	CCACCAGATGGGAGTAGAC	1 cycle (95°C for 1 min), 45 cycles (95°C for 5 s, 60°C for 10 s)	118 bp	Kim et al., 2022
	Meg 1139R	GGTTGATATTGCCCATGTCCA			
	Meg 1079P	[FAM]CCTACTA[i‐EBQ]CTTTGCGCCCA GCATG[phosphate]			
*Pst* I fragment	1F	CTCAAACACTCTGGCTCATC	1 cycle (94°C for 5 min), 30 cycles (94°C for 30 s, 58°C for 1 min, 72°C for 1 min), 72°C for 5 min	570 bp	Kurita et al., 1998
	1R	GCACCAACACATCTCCTATC			
DNA polymerase	4F	CGGGGGCAATGACGACTACA		568 bp	Oseko et al., 2004
	4R	CCGCCTGTGCCTTTTCTGGA			

Statistical analyses were performed using GraphPad Prism 10 (GraphPad Inc., USA). Survival data were analysed using Kaplan–Meier survival analysis, and statistical significance was determined using the log‐rank (Mantel–Cox) test. For viral load data obtained from immersion challenges, differences across time within each group were analysed using one‐way analysis of variance (ANOVA) followed by Dunnett's multiple comparison test, with the initial virus detection time point used as the control. For cohabitation experiments, viral load data were analysed using two‐way ANOVA with time and group (donor vs. recipient) as factors, including only the time points at which both groups were measured. Multiple comparisons were performed using Šídák's multiple comparisons test. Viral shedding data (copies/L/g) from both immersion and cohabitation experiments were analysed using one‐way ANOVA followed by Dunnett's multiple comparison test, with the initial virus detection time point used as the control. Statistical significance is indicated as follows: *p < 0.05, **p < 0.01, ***p < 0.001 and ****p < 0.0001.

At a concentration of 10^5^ copies/mL, black sea bream exhibited a 90% survival rate, whereas none of the Japanese sea bass individuals survived (0%). At a concentration of 10^3^ RSIV copies/mL, black sea bream demonstrated a survival rate of 96.6% and Japanese sea bass 60% (Figure 1a,b). The control group exhibited a survival rate of 100%. In cohabitation trials, donor black sea bream individuals exhibited survival rates of 90%, 93.4% and 36.6% when paired with recipient black sea bream individuals, Japanese sea bass and rock bream, respectively. Among recipient fish exposed to infected black sea bream, the survival rate was 93.4% for black sea bream, whereas Japanese sea bass and rock bream experienced 0% survival (Figure 1c–e). In contrast, donor Japanese sea bass demonstrated 0% survival. In recipient groups co‐cultured with infected Japanese sea bass, survival reached 90% for black sea bream but remained 0% for Japanese sea bass and rock bream (Figure 1f–h). The control group maintained a survival rate of 100% throughout the experimental period.

**FIGURE 1:**
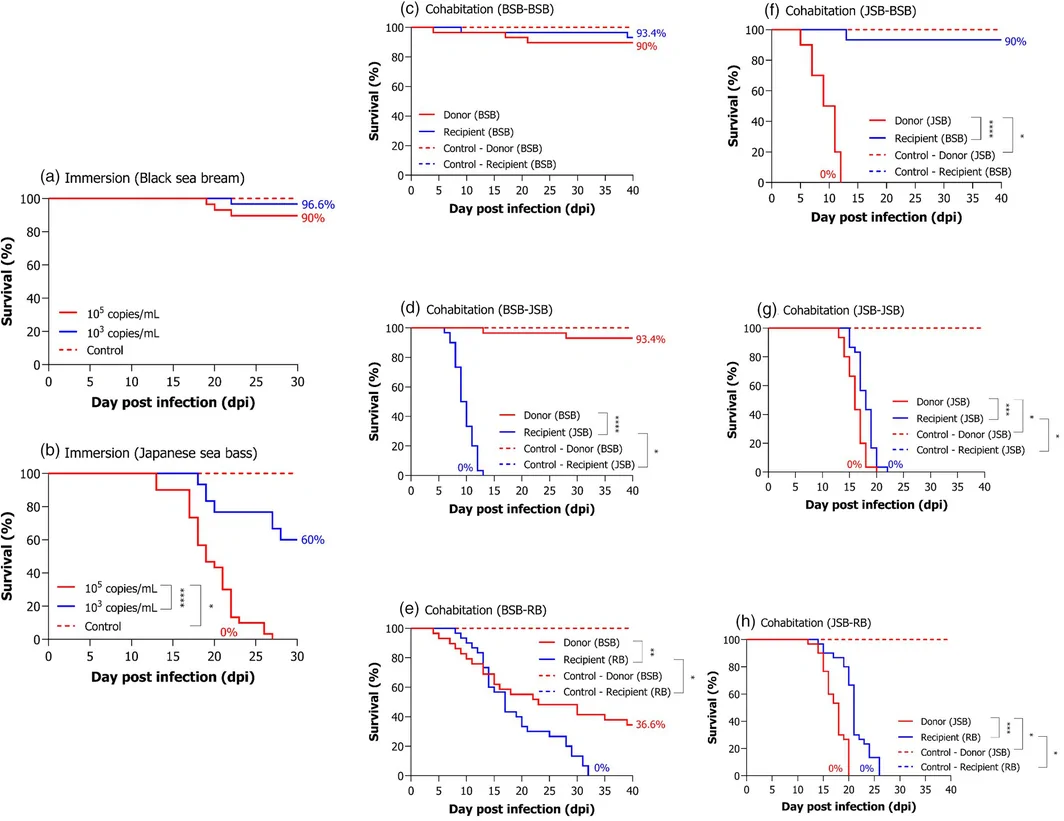
Survival and cumulative mortality of marine finfish after immersion and cohabitation with RSIV challenges. (a, b) Fishes were exposed to RSIV at concentrations of 10^3^ or 10^5^ copies/mL by immersion for 4 h at 25°C, followed by complete water exchange, and monitored for 30 days (*n* = 30 fish per group). (a) Black sea bream immersed with 10^3^ or 10^5^ copies/mL RSIV. (b) Japanese sea bass immersed with 10^3^ or 10^5^ copies/mL RSIV. No mortality was observed in the control groups. (c, e) Cumulative mortality of naive recipient fish after cohabitation with RSIV‐infected donor black sea bream at 25°C. Donor fish were intraperitoneally injected with 10^6^ RSIV copies/fish, whereas recipients received no treatment. Recipient species were (c) black sea bream, (d) Japanese sea bass and (e) rock bream. (f, h) Cumulative mortality of naive recipient fish after cohabitation with RSIV‐infected donor Japanese sea bass at 25°C. Donor fish were intraperitoneally injected with 10^6^ RSIV copies/fish, whereas recipients received no treatment. Recipient species were (f) black sea bream, (g) Japanese sea bass (g) and (h) rock bream. Control donor fish were intraperitoneally injected with 100 μL of virus‐free L‐15 medium, and no mortality was observed in any control group throughout the experimental period. Survival curves were analysed using the Kaplan–Meier method, and statistical significance was determined using the log‐rank (Mantel–Cox) test. Significant differences are as follows: *p < 0.05, **p < 0.01, ***p < 0.001, ****p < 0.0001; ns, not significant, is omitted. BSB, black sea bream; JSB, Japanese sea bass; RB, rock bream; RSIV, red sea bream iridovirus.

In black sea bream exposed to 10^5^ copies/mL of RSIV, viral loads increased from 7 dpi, coinciding with the initial detection of viral shedding. Japanese sea bass exhibited a similar increase in viral loads, with detectable viral shedding until 21 dpi (****p < 0.0001). At a concentration of 10^3^ copies/mL, black sea bream maintained low viral loads throughout the experimental period, with no detectable shedding. In contrast, Japanese sea bass exhibited increasing viral loads accompanied by sustained viral shedding until 21 dpi (Figure 2a,b). In cohabitation experiments in which black sea bream served as donors, viral loads in donor fish increased between 4 and 7 dpi (****p < 0.0001) and subsequently declined, with viral shedding exhibiting a comparable temporal trend (Figure 2c,d). Recipient rock bream and Japanese sea bass exhibited detectable viral loads before mortality, whereas recipient black sea bream demonstrated comparatively lower peak viral loads. When Japanese sea bass were used as donors, viral loads increased rapidly preceding donor mortality, and viral shedding followed a similar pattern (Figure 2e,f). In the corresponding recipient groups, rock bream and Japanese sea bass demonstrated significant increases in viral loads, culminating in complete mortality. In contrast, the recipient black sea bream exhibited only transient viral detection, followed by a decline in viral loads and viral shedding.

**FIGURE 2:**
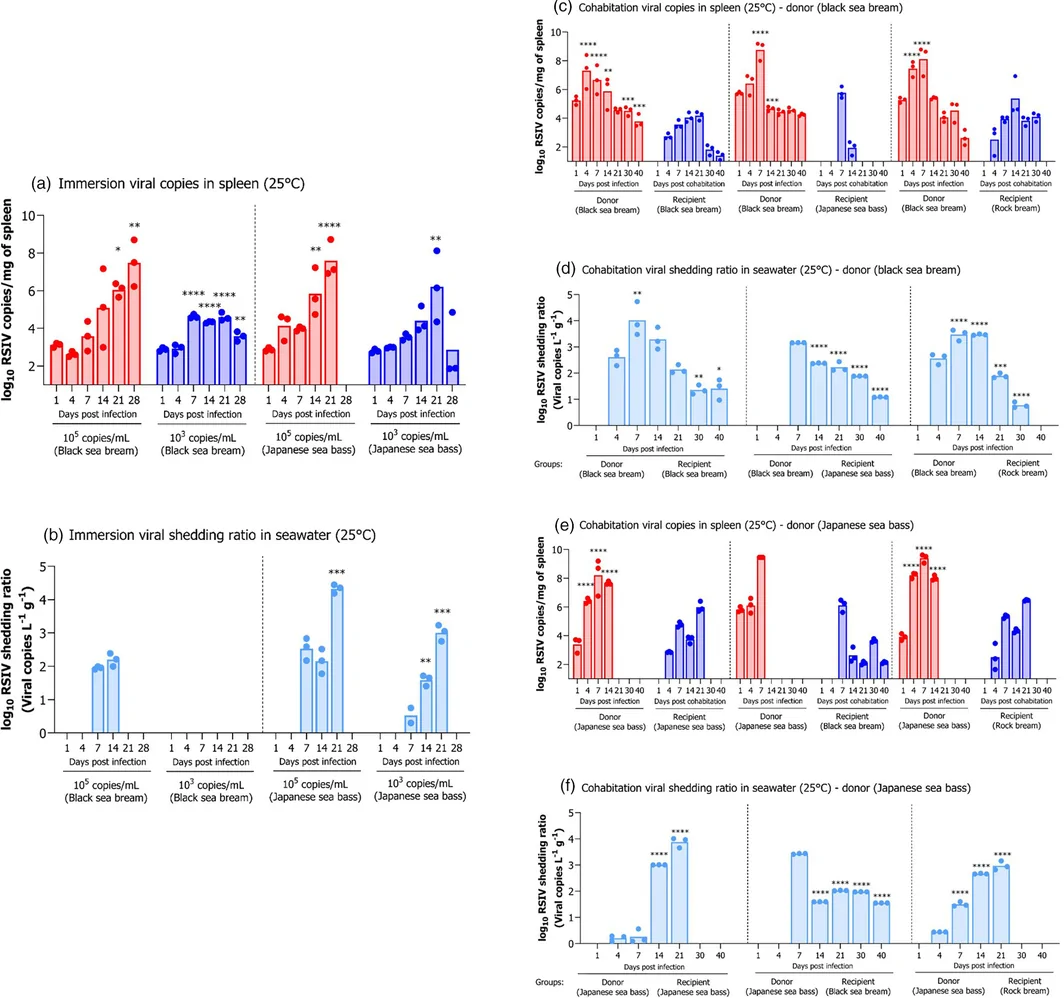
Viral loads in spleen and viral shedding ratios after immersion and cohabitation challenges with RSIV. (a, b) Immersion challenge performed at 25°C, in which fish were exposed to RSIV at concentrations of 10^3^ or 10^5^ copies/mL for 4 h, followed by complete water exchange, and sampled for up to 30 days (*n* = 3 fish per time point). (a) Viral loads measured in spleen tissues of black sea bream and Japanese sea bass after immersion challenge and (b) viral shedding ratios detected in the rearing seawater of black sea bream and Japanese sea bass after immersion challenge. (c, d) Cohabitation challenge using RSIV‐infected donor black sea bream, which were intraperitoneally injected with 10^6^ RSIV copies/fish at 25°C, with naive recipients receiving no treatment. (c) Viral loads measured in spleen tissues and (d) viral shedding ratios detected in the corresponding rearing seawater. (e, f) Cohabitation challenge using RSIV‐infected donor Japanese sea bass, which were intraperitoneally injected with 10^6^ RSIV copies/fish at 25°C. (e) Viral loads measured in spleen tissue and (f) viral shedding ratio detected in the corresponding rearing seawater. Viral shedding ratios were calculated as viral genome copy numbers per litre of seawater normalised by the total biomass (g) of remaining fish in each tank (copies/L/g). RSIV copy numbers were analysed in three fish and the corresponding seawater samples at each sampling interval. Viral load data from immersion challenges were analysed using one‐way analysis of variance (ANOVA) followed by Dunnett's multiple comparison test, with the initial virus detection time point within each group used as the control. Viral load data from cohabitation experiments were analysed using two‐way ANOVA with Šídák's multiple comparisons test, using only shared time points between donor and recipient groups. Viral shedding data were analysed using one‐way ANOVA followed by Dunnett's multiple comparison test, with the initial virus detection time point used as the control. Statistical significance is indicated as follows: *p < 0.05, **p < 0.01, ***p < 0.001, ****p < 0.0001; ns, not significant, is omitted. RSIV, red sea bream iridovirus.

Not only in South Korea but also worldwide, cage‐type aquaculture systems often rear multiple fish species within the same waters, and this arrangement can lead to pathogen transmission through the rearing environment (Jahangiri et al., 2022; Kim et al., 2026; Mukherjee et al., 2020). Therefore, this study explored the potential risk of RSIV transmission between Japanese sea bass and black sea bream through a cohabitation experiment mimicking the aquaculture environment.

Immersion infection has been widely adopted as an experimental infection model in pathogenicity research, due to its capacity to replicate, under controlled laboratory settings, disease dynamics that occur in natural settings (Hu et al., 2024; Huang et al., 2026). In the present study, we used immersion infection to characterise the infection dynamics of RSIV in black sea bream and Japanese sea bass. Although viral replication was confirmed in black sea bream after immersion challenge, mortality remained low. This pattern may reflect decreased infectivity due to factors such as the target receptor range of the virus at the cellular level, infection efficiency or viral maturation. The mechanisms governing viral entry into cells and their intracellular trafficking vary depending on receptor binding specificity (Mercer et al., 2010). Even when DNA viral genome replication proceeds actively, host‐mediated control mechanisms can partially suppress the viral assembly, maturation and release stages, reducing the proportion of infectious particles produced (Zenner et al., 2013). These findings suggest that host cell specificity influences viral entry, replication and infectious particle production. This interpretation is supported by evidence that the same virus may exhibit varying degrees of replication depending on the type of infected tissue or cell (Gibson‐Kueh et al., 2003). Therefore, the high viral loads coupled with low pathogenicity observed in black sea bream may reflect differences in binding specificity or infectivity at the cellular level rather than the absence of productive infection. Further analysis of the infection pathway of RSIV in this species at the cellular level is necessary to elucidate this mechanism.

In the present study, under the same immersion infection conditions, Japanese sea bass exhibited a different infection pattern compared to black sea bream. The 10^5^ copies/mL group exhibited significantly elevated viral loads and viral shedding rates after 21 dpi, with all individuals dying by 27 dpi. In contrast, in the 10^3^ copies/mL group, the viral loads decreased by 28 dpi, and 40% of individuals survived. These results suggest that RSIV infectious particles replicated more efficiently in Japanese sea bass than in black sea bream. A previous study using a sea perch fry cell line revealed that iridovirus replication depends on host cell signalling pathways and cycle regulation mechanisms, with these responses determining replication efficiency (Wang et al., 2023). In this context, the high infectivity and mortality rate observed in Japanese sea bass suggest that host cells in this species provide an intracellular environment conducive to efficient viral replication. Conversely, when the initial infection dose is low, a proportion of infected cells fail to undergo productive viral replication (Howell et al., 2024). This phenomenon is consistent with the reduced viral load observed in the low‐concentration infection group in the present study, suggesting that the initial infection concentration may influence infection persistence. Therefore, the severity of RSIV‐associated disease in Japanese sea bass may vary depending on the concentration of RSIV present in the rearing water.

In our study, the group with black sea bream as the donor maintained a low mortality rate despite exhibiting high viral loads. Conversely, the recipient species, rock bream and sea bass, exhibited high viral loads during the active mortality phase, and all individuals in these groups had died by the end of the experiment. However, in the recipient black sea bream, viral loads increased until 21 dpi before subsequently decreasing, accompanied by a high survival rate. These results indicate that although the black sea bream were infected, mortality was low, suggesting the possibility of subclinical or asymptomatic infection. However, the presence of infectious viral particles in the tissues was not confirmed in this study, because infectivity assays such as virus isolation using cell culture were not performed. Similar interspecies transmission has been reported for Cyprinid herpesvirus‐3 between goldfish and carp (El‐Matbouli & Soliman, 2011). Therefore, further studies are required to determine whether black sea bream can serve as a carrier or reservoir host. Reservoir hosts maintain pathogens internally while exhibiting low disease expression, contributing to the persistent transmission of pathogens within a shared environment (Brook & Dobson, 2015). This suggests that in environments where multiple fish species cohabitate within the same waters, black sea bream may act as a source of RSIV transmission. In contrast, Japanese sea bass donors exhibited high infectivity and complete mortality, whereas recipient fish (rock bream, Japanese sea bass and black sea bream) exhibited infection patterns consistent with those observed when black sea bream served as the donor. These results indicate that, unlike black sea bream, Japanese sea bass may act as amplifying hosts through high infectivity and rapid mortality, thereby facilitating transmission to surrounding individuals. This distinction aligns with prior studies indicating that in multi‐host infection systems, different host populations contribute differently to pathogen maintenance and transmission, with certain species potentially acting as major sources (Haydon et al., 2002). Collectively, our results demonstrate that in shared marine areas, such as aquaculture sites, Japanese sea bass and black sea bream may play distinct but complementary epidemiological roles in the transmission of RSIV.

However, this study measured viral loads only in the spleen and viral shedding ratios, and therefore the viral infection cycle at the cellular level could not be directly confirmed. In addition, histopathological analyses were not performed, which limits our ability to fully explain species‐specific differences in infectivity and disease progression. Furthermore, although immersion infection and cohabitation experiments were designed to mimic aquaculture conditions, these controlled laboratory settings may not fully capture the environmental complexity of field aquaculture systems. Future studies incorporating cellular‐level analyses, histopathological validation and transmission experiments under field‐relevant environmental conditions will be necessary to better clarify the epidemiological roles of different host species and to improve understanding of RSIV transmission dynamics in aquaculture ecosystems. Overall, our findings provide experimental evidence that Japanese sea bass and black sea bream may play distinct yet complementary roles in RSIV maintenance and spread within shared marine farming environments. The present study also has implications for the management of RSIV in net‐pen aquaculture systems where multiple species are cultured in close proximity. Because viral loads were detected in black sea bream despite low mortality, this species may act as a potential source of virus release, indicating that routine monitoring may be necessary even in the absence of clinical signs. In addition, detection of viral shedding into seawater suggests that environmental monitoring may be useful for early detection of RSIV circulation in aquaculture farms. Because interspecies transmission was confirmed under cohabitation conditions, management strategies such as increasing the distance between net pens or avoiding co‐culture of susceptible and carrier species may help reduce the risk of RSIV outbreaks.

## AUTHOR CONTRIBUTIONS

**Chae‐yeong Ji:** conceptualisation, methodology, validation, formal analysis, investigation, data curation, visualisation, writing – original draft, writing – review and editing. **Sung‐Bin Moon:** conceptualisation, investigation, formal analysis. **Jae‐Ok Kim:** supervision, project administration, funding acquisition. **Mun‐Gyeong Kwon:** supervision, project administration, funding acquisition. **Chan‐Il Park:** conceptualisation, funding acquisition, data curation, supervision, resources, writing – review and editing. **Kyung‐Ho Kim:** conceptualisation, methodology, validation, formal analysis, investigation, data curation, visualisation, writing – original draft, writing – review and editing, funding acquisition, supervision, resources.

## FUNDING INFORMATION

This study was supported by a grant from the National Fishery Products Quality Management Service (Development of Quarantine and Disease Control Program for Aquatic Life, NFQS 2026001).

## CONFLICT OF INTEREST STATEMENT

The authors declare no conflicts of interest.

## Data Availability

The data that support the findings of this study are available from the corresponding author upon reasonable request.
